# Analytical Method Development and Validation for the Quantification of Acetone and Isopropyl Alcohol in the Tartaric Acid Base Pellets of Dipyridamole Modified Release Capsules by Using Headspace Gas Chromatographic Technique

**DOI:** 10.1155/2018/8240932

**Published:** 2018-03-01

**Authors:** Sriram Valavala, Nareshvarma Seelam, Subbaiah Tondepu, V. Shanmukha Kumar Jagarlapudi, Vivekanandan Sundarmurthy

**Affiliations:** ^1^Department of Chemistry, K L University, Green Fields, Vaddeswaram, Guntur 522502, Andhra Pradesh, India; ^2^Research and Development, Bluefish Pharmaceuticals Private Limited, Bangalore 560115, Karnataka, India

## Abstract

A simple, sensitive, accurate, robust headspace gas chromatographic method was developed for the quantitative determination of acetone and isopropyl alcohol in tartaric acid-based pellets of dipyridamole modified release capsules. The residual solvents acetone and isopropyl alcohol were used in the manufacturing process of the tartaric acid-based pellets of dipyridamole modified release capsules by considering the solubility of the dipyridamole and excipients in the different manufacturing stages. The method was developed and optimized by using fused silica DB-624 (30 m × 0.32 mm × 1.8 *µ*m) column with the flame ionization detector. The method validation was carried out with regard to the guidelines for validation of analytical procedures Q2 demanded by the International Council for Harmonisation of Technical Requirements for Pharmaceuticals for Human Use (ICH). All the validation characteristics were meeting the acceptance criteria. Hence, the developed and validated method can be applied for the intended routine analysis.

## 1. Introduction

**Figure 1:**
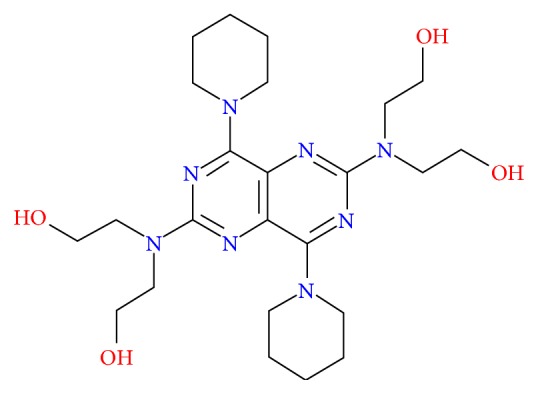
Structure of dipyridamole.

Dipyridamole (2,6-bis-(diethanolamino)-4,8-dipiperidino-(5,4-d)-pyrimidine) displays antithrombotic and antiaggregatory activity. The dipyridamole (Figure 1) is used in combination with “blood thinners” such as warfarin to avoid clot formation after heart valve replacements. Clots are a serious complication that can cause strokes, heart attacks, or blocked blood vessels in the lungs (pulmonary embolisms). Dipyridamole is an antiplatelet drug. Dipyridamole is an odourless yellow crystalline powder, having a bitter taste.

Dipyridamole exhibits a relatively short biological half-life of less than one hour. Therefore, extended release formulations of dipyridamole, which provide a continual administration of active ingredient over time, are preferred. Dipyridamole is soluble in acidic mediums with a pH below 4 and is practically insoluble in water. Therefore, dipyridamole is readily absorbed in the more acidic regions of the upper gastrointestinal tract, but remains insoluble in the more basic regions of the intestine. To obtain a constant level of dipyridamole in the blood, it is advantageous to formulate a dipyridamole dosage form that releases dipyridamole at a controlled rate and at a defined pH. Acidic components can be coadministered with dipyridamole to maintain a defined pH level throughout administration. Dipyridamole can also be administered with other active ingredients, such as aspirin. Aspirin (acetylsalicylic acid) is an inhibitory substance which counteracts the aggregation of human blood platelets by inhibiting cyclooxygenase and thereby inhibiting the biosynthesis of the aggregation promoting thromboxane A2 [1, 2]. The residual solvents present in tartaric acid-based pellets of dipyridamole modified release capsules are classified as class 3 solvents as per the ICH Q3C guidelines.

The dipyridamole in tartaric acid-based pellets of dipyridamole modified release 150 mg and 200 mg capsules are available in the market. Each capsule contains dipyridamole 200 mg and 150 mg respective dosage strength. The adults including the elders recommended dose is one capsule twice daily, usually one in the morning and one in the evening preferably with meals. The capsules should be swallowed whole without chewing as per eMC [3].

In the literature survey, quite a few GC method have been reported from the determination of the residual solvents in dipyridamole API [4], few liquid chromatographic methods have been reported for determination of dipyridamole in pharmaceutical preparation [4–6], and few methods have been reported for dipyridamole and its degradation product [7, 8]. However, several methods were reported for determination of dipyridamole in combination with other drugs [9–12]. Estimation of dipyridamole and its metabolites in human plasma by liquid chromatographic-mass spectroscopy and HPLC has been performed [13–15].

The aim of this study is to develop the simple and fast analytical method for estimation of residual solvents in the tartaric acid-based pellets of dipyridamole modified release capsules, and the method can be used for the routine analysis. The developed method was subjected for the analytical validation with respect to specificity, linearity, precision, accuracy, limit of detection (LOD), limit of quantification (LOQ), robustness, and ruggedness as per the ICH guidelines [16].

## 2. Materials and Methods

### 2.1. Chemicals and Reagents

The GC grade N,N-dimethylsulfoxide, isopropyl alcohol, acetone, HPLC grade water, nitrogen gas, air, and hydrogen. The dipyridamole drug substance, placebo samples of dipyridamole modified release capsules, and samples of dipyridamole modified release capsules were supplied by Bluefish Pharmaceuticals Pvt. Ltd, Bangalore, India.

### 2.2. Equipment

The analytical method was developed by using the Agilent 7890A coupled with G1888 network headspace sampler, analytical balance from Mettler Toledo, micropipette from Eppendorf, headspace crimp vials, and suitable glass apparatus for solution preparations.

### 2.3. Chromatographic Conditions

The method was developed and validated by using fused silica DB-624 (30 m × 0.32 mm × 1.8 *µ*m) column with the flame ionization detector (FID) and the chromatographic parameters are given in Table 1. The chromatographic retention time of acetone and isopropyl alcohol is given in Table 2.

### 2.4. Preparation of Solutions

#### 2.4.1. Diluent Solution

A mixture of N,N-dimethylsulfoxide and water was used as diluent.

#### 2.4.2. Blank Solution

Transfer 5 mL of diluent into 20 mL headspace and crimp cap immediately.

#### 2.4.3. Preparation of Standard Solution (Stock)

Weigh and transfer about 500 mg of isopropyl alcohol and 500 mg of acetone into 50 mL volumetric flask containing about 30 mL of diluent mix and make up to the mark with diluent.

#### 2.4.4. Preparation of Standard Solution

Pipette out 5 mL of the above standard stock solution into 100 mL volumetric flask and make up to the mark with diluent. Transfer 5 mL of above solution into 20 mL headspace vials, and crimp cap immediately.

#### 2.4.5. Preparation of Sample Solution

Open five capsules and crush the pellets using mortar pestle. Weigh and transfer 500 mg of crushed powder into 20 mL headspace vial, add 5 mL of diluent, and crimp the cap immediately.

### 2.5. System Suitability Criteria

The present relative standard deviation of standard peak area for six replicate injections should not be more than 10.

## 3. Results and Discussion

### 3.1. Method Development and Optimization

The analytical method development was initiated by using the Agilent 7890A coupled with G1888 network headspace sampler, fused silica DB-624 (30 m × 0.32 mm × 1.8 *µ*m) column with the flame ionization detector (FID), carrier gas as helium. The front injector conditions (injector temperature 140°C, carrier gas flow 2.0 mL/min, and split ration 5 : 1), front detector conditions (detector temperature 260°C, hydrogen flow 40 mL/minute, and air flow 300 mL/minute), oven conditions (40°C for 10 minutes and increasing the temperature to 250°C at the rate of 30°C/minute and hold for 10 minutes), headspace oven temperature 100°C, loop temperature 105°C, transfer line temperature 110°C. The dimethylsulfoxide used as diluent for solution preparations. Based on the above experiment, we found the very less resolution between acetone and isopropyl alcohol. The further experiments were conducted by altering the chromatographic conditions to achieve satisfactory resolution between acetone and isopropyl alcohol.

The further experiment was conducted by using the above experiment chromatographic parameters and by changing the front injector condition and headspace sampler condition. Based on the above experiment, we found the very less resolution between acetone and isopropyl alcohol and found the placebo peak interference was observed at the retention time of acetone. The aim is to resolve the issue of placebo peak interference, needs to modify the diluent or headspace oven temperature conditions to finalize the method conditions.

The further experiment was conducted by using the fused silica DB-624 (30 m × 0.32 mm × 1.8 *µ*m) column with the flame ionization detector (FID), carrier gas as nitrogen. The front injector conditions (injector temperature 140°C, carrier gas flow 1.0 mL/min, and split ration 20 : 1), front detector conditions (detector temperature 260°C, hydrogen flow 30 mL/minute, and air flow 300 mL/minute), oven conditions (40°C for 10 minutes and increasing the temperature to 210°C at the rate of 35°C/minute and hold for 5 minutes), headspace oven temperature 80°C, loop temperature 90°C, transfer line temperature 100°C. The misture of N,N-dimethylsulfoxide and water used as diluent for solution preprations. Based on the above experiment, it was found that no placebo interference was observed at the retention time of acetone and isopropyl alcohol, and resolution was found satisfactory.

Based on the optimization of the trials, the abovementioned chromatographic conditions were finalized for the quantification of the acetone and isopropyl alcohol in tartaric acid-based pellets of dipyridamole modified release capsules. Hence, this method can be validated and introduced for the routine analysis.

### 3.2. Method Validation

The developed analytical method for quantification of the residual solvents in the tartaric acid-based pellets of dipyridamole modified release capsules was validated as per International Council for Harmonisation of Technical Requirements for Pharmaceuticals for Human Use (ICH) [15]. The validation parameters [17, 18] specificity, estimation of limit of detection (LOD), and limit of quantification (LOQ), accuracy, precision, linearity, range, ruggedness, and robustness were examined [17, 18].

#### 3.2.1. System Suitability

To check the system suitability criteria, the solutions were prepared and injected as per the test method. All the parameters were found well within the acceptance criteria (Table 3).

#### 3.2.2. Limit of Detection and Limit of Quantification

The limit of detection (LOD) and limit of quantification (LOQ) were established by the signal-to-noise ratio method by preparing the known concentrations of acetone and isopropyl alcohol and injected into gas chromatography headspace instrument as per the test method. The limit of detection and limit of quantification for each matrix were determined from the signal-to-noise ratio (S/N) method of 3 : 1 and 10 : 1 by injecting the standard solutions (Table 4). The LOD and LOQ were verified by analysis of spiked standard solutions predefined acceptance criteria. The percent relative standard deviation for area response found for acetone at LOD concentration is 7.1 and LOQ is 3.5 and for isopropyl alcohol at LOD concentration is 5.2 and LOQ is 3.7.

#### 3.2.3. Specificity

Specificity was accomplished by injecting the samples as per the test method and as a part of the specificity study. Blank, acetone, isopropyl alcohol solvent, and placebo were prepared and injected as per test method. No peak interference at the retention time of acetone and isopropyl alcohol was observed. Therefore, we conclude that this method is selective and suitable for the identification and quantification of the acetone and isopropyl alcohol in the dipyridamole modified release capsules.

**Figure 2:**
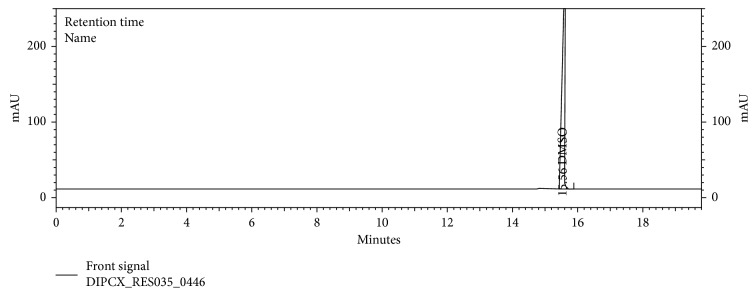
Typical chromatogram of blank.

**Figure 3:**
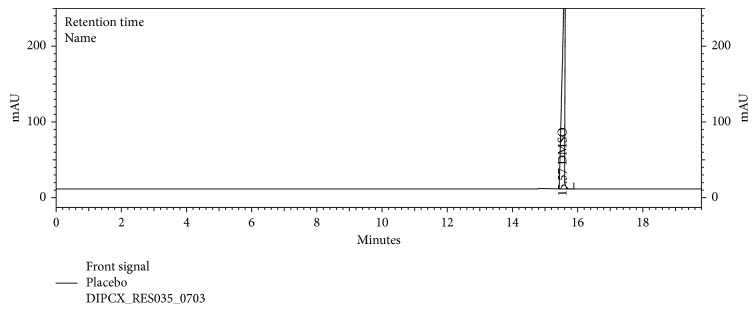
Typical chromatogram of placebo.

**Figure 4:**
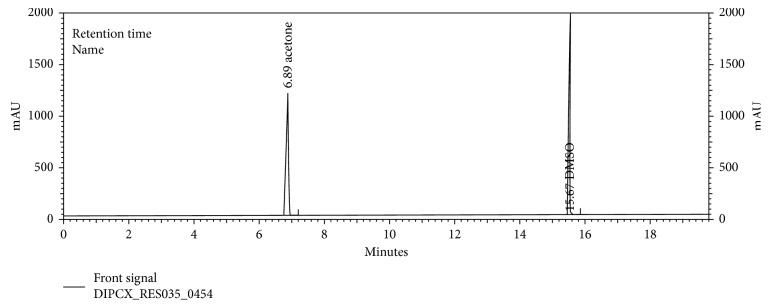
Typical chromatogram of acetone.

**Figure 5:**
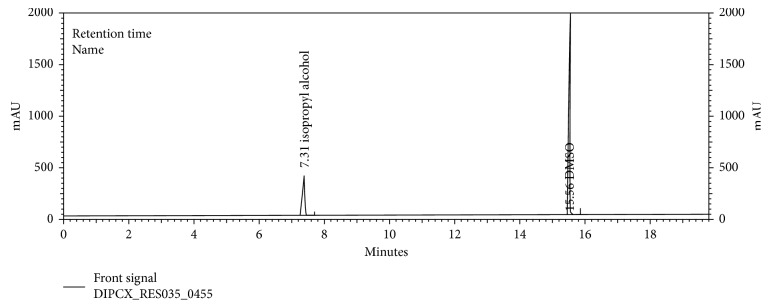
Typical chromatogram of isopropyl alcohol.

**Figure 6:**
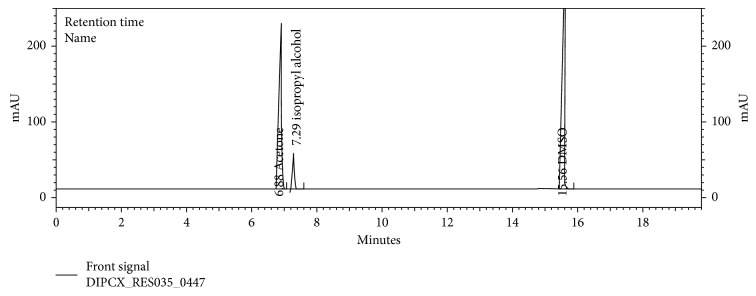
Typical chromatogram of standard solution.

**Figure 7:**
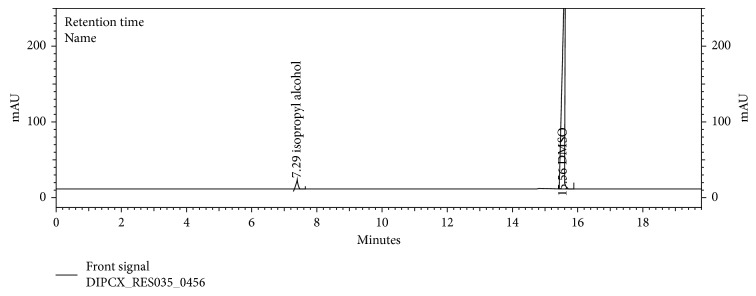
Typical chromatogram of as-such sample.

**Figure 8:**
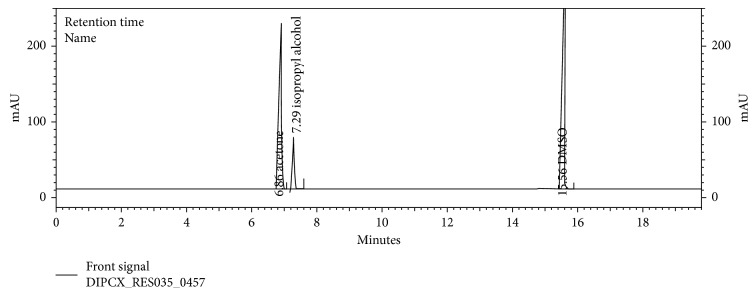
Typical chromatogram of spiked sample.

The chromatograms are given for the blank (Figure 2), placebo (Figure 3), acetone (Figure 4), isopropyl alcohol (Figure 5), standard (Figure 6), as-such sample (Figure 7), and spiked blend solution (Figure 8).

#### 3.2.4. Method Precision (Repeatability)

**Figure 9:**
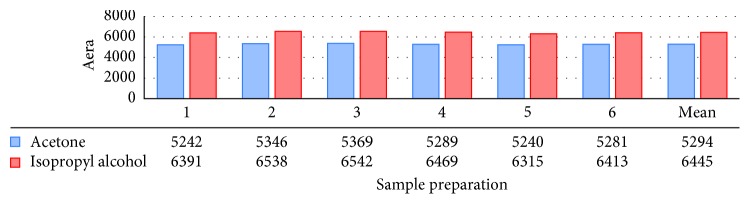
Method precision.

The method precision or intraassay precision was performed by preparing the six replicate test preparations (*n*=6) of dipyridamole 200 mg modified release capsules by spiking acetone and isopropyl alcohol specification level (Table 5 and Figure 9) analyzed as per the test method. The concentration in parts per million was calculated and found to be within the acceptance criteria. The relative standard deviations obtained for acetone were 1.0% and isopropyl alcohol 1.4%. The graphs for acetone and isopropyl alcohol are shown in Figure 9.

#### 3.2.5. Accuracy

**Figure 10:**
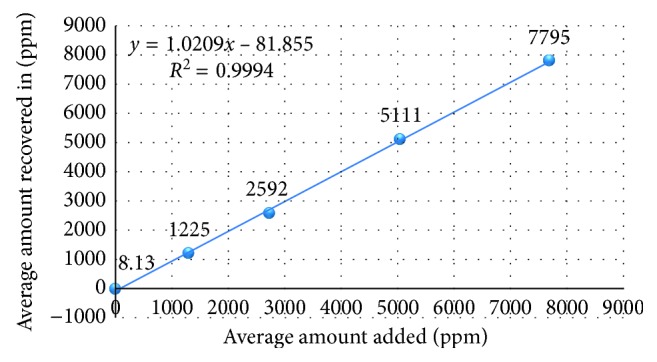
Linearity plot for acetone from accuracy results.

**Figure 11:**
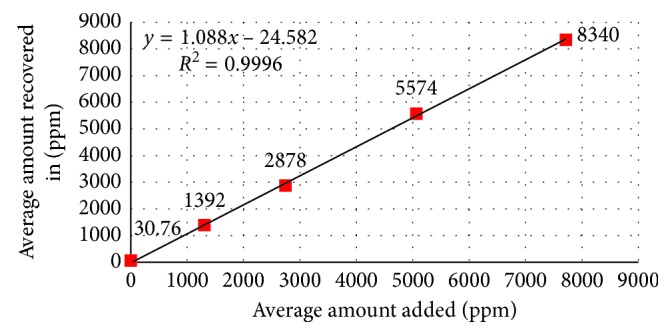
Linearity plot for isopropyl alcohol from accuracy results.

Accuracy of the proposed analytical procedure was evaluated from the assay results of the acetone and isopropyl alcohol as per the test method. A series of sample solutions were prepared in triplicate (six replicate test preparations for LOQ and about 200% levels) by spiking the acetone and isopropyl alcohol in placebo except LOQ level in the range of about 25%, 50%, 100%, and 150% of specification level and injected into HPLC system and analyzed as per the test method. The concentrations of acetone are 7.5 *µ*g/mL, 1300 *µ*g/mL, 2738 *µ*g/mL, 5050 *µ*g/mL, and 7694 *µ*g/mL and of isopropyl alcohol are 28 *µ*g/mL, 1304 *µ*g/mL, 2745 *µ*g/mL, 5063 *µ*g/mL, and 7715 *µ*g/mL. Individual % recovery, mean % recovery, % RSD, and squared correlation coefficient for linearity of the test method were calculated, and the results were found to be within the acceptance criteria (Table 6). The linearity graphs from accuracy results for acetone and isopropyl alcohol are shown in Figures 10 and 11, respectively.

#### 3.2.6. Linearity

The linearity was studied by analyzing the standard solutions. A series of solutions of acetone and isopropyl alcohol solutions were prepared in the range of LOQ to about 150% of specification level and injected into the HPLC system. Linearity of detector response was established by plotting a graph between concentration versus response of acetone and isopropyl alcohol peaks. The detector response was found to be linear from about LOQ to 150% of specification level and injected into HPLC system and analyzed as per the test method. The concentrations of acetone are 7 *µ*g/mL, 1260 *µ*g/mL, 3024 *µ*g/mL, 5040 *µ*g/mL, and 7560 *µ*g/mL and of isopropyl alcohol are 27 *µ*g/mL, 1261 *µ*g/mL, 3027 *µ*g/mL, 5045 *µ*g/mL, and 7567 *µ*g/mL.

**Figure 12:**
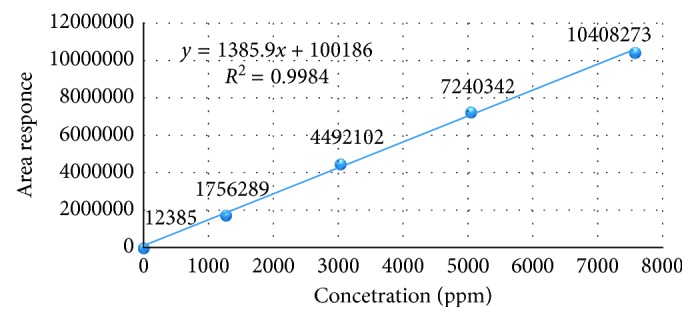
Linearity plot for acetone.

**Figure 13:**
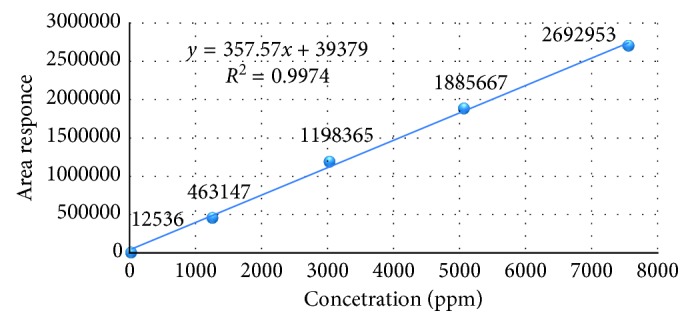
Linearity plot for isopropyl alcohol.

The square of correlation coefficient, slope, and % *y*-intercept at 100% level, intercept, and residual sum of squares were calculated, and the results were found to be within the acceptance criteria (Table 7).The linearity graphs from accuracy results for acetone and isopropyl alcohol are shown in Figures 12 and 13, respectively.

#### 3.2.7. Ruggedness (Intermediate Precision)

Intermediate precision was performed by preparing the six replicate test preparations (*n*=6) of dipyridamole 200 mg modified release capsules by spiking acetone and isopropyl alcohol at specification level and analyzed as per the test method by using different Headspace gas chromatography system, different column of same make by different analyst on different day. The concentration in parts per million was calculated and found to be within the acceptance criteria. The overall concentration in parts per million for replicate preparations (*n*=12) of method precision and intermediate precision was calculated and found to be within the acceptance criteria (Table 8). The relative standard deviations obtained for acetone was 1.4% and isopropyl alcohol was 3.1%.

#### 3.2.8. Solution Stability

The solution stability of acetone and isopropyl alcohol was determined by keeping sample solution and standard solutions at room temperature for 1 day and 2 day and measured against freshly prepared standard solution. The standard solution and sample solutions were found stable for 2 days at room temperature.

#### 3.2.9. Robustness

Robustness of the proposed method was performed by keeping the chromatographic conditions constant with the following deliberate variations:

- Change in carrier gas flow rate
- Change in column oven temperature
- Change in headspace sampler vial equilibration time
- Change in headspace vial oven temperature

**Figure 14:**
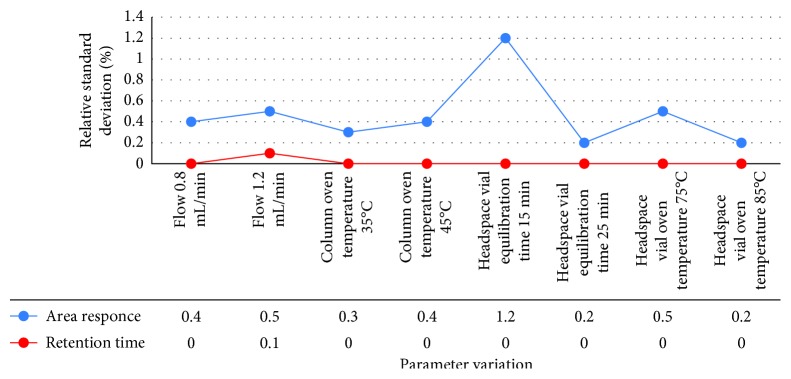
Robustness data for acetone.

**Figure 15:**
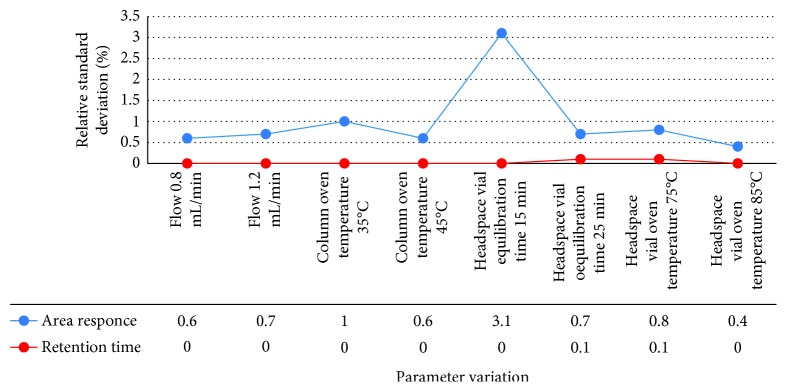
Robustness data for isopropyl alcohol.

The standard solution was injected six times in replicate for each abovementioned change. The system suitability parameters like % relative standard deviation for area response and % relative standard deviation for retention time were recorded for acetone and isopropyl alcohol and found well within the acceptance criteria. The results are given in Table 9, and the graphs for acetone and isopropyl alcohol are shown in Figures 14 and 15, respectively.

#### 3.2.10. Application of the Proposed Method

The developed analytical method was applied to the analysis of real samples from the manufacturing unit. All the analytical validation parameters could be confirmed, and the method was proven to be suitable for routine analysis regarding rapid and accurate results.

## 4. Conclusions

A simple, sensitive, accurate, robust headspace gas chromatographic method was developed for the quantitative determination of residual solvents in tartaric acid-based pellets of dipyridamole modified release capsules. The proposed method was validated and found to be precise, accurate, linear, robust, and rugged, and all the validation parameter results were found satisfactory. The described method is suitable for routine analysis of production samples at laboratories.

## Figures and Tables

**Table 1:** Chromatographic parameters.

Column	Fused silica DB-624 (30 m × 0.32 mm × 1.8 *µ*m)	Makeup gas flow	25 mL/minute
Detector	Flame ionization detector (FID)	Oven temperature	40°C for 10 minutes and increasing the temperature to 210°C at the rate of 35°C/minute and kept for 5 minutes
Mode	Constant flow	*Headspace sampler condition*	
Carrier gas	Nitrogen	Oven temperature	80°C
*Front Injector condition*		Loop temperature	90°C
Inject temperature	140°C	Transfer line temperature	100°C
Carrier gas flow	1.0 mL/min	GC cycle time	34 minutes
Split ratio	20 : 1	Vial equilibration time	20 minutes
Injector type	Split	Pressurization time	1.0 minutes
*Front detector condition*		Loop fill time	0.2 minutes
Detector temperature	260°C	Loop equilibration time	0.05 minutes
Hydrogen flow	30 mL/minute	Injection time	1.0 minutes
Air flow	300 mL/minute	Shake	High

**Table 2:** Retention times of acetone and isopropyl alcohol.

Serial Number	Impurity name	RT (about)
1	Acetone	6.8
2	Isopropyl alcohol	7.3

**Table 3:** System suitability criteria and results

Parameter	Acceptance criteria	Result
The present relative standard deviation of acetone peak area for six replicate injections.	≤10.0	0.9
The present relative standard deviation of isopropyl alcohol peak area for six replicate injections.	≤10.0	1.3
The present relative standard deviation of acetone retention time for six replicate injections.	≤10.0	0.0
The present relative standard deviation of isopropyl alcohol retention time for six replicate injections.	≤10.0	0.0

**Table 4:** Limit of detection and Limit of quantification.

Name	LOD	LOQ
	Concentration (*µ*g/mL)	Concentration (*µ*g/mL)
Acetone	3	7
Isopropyl alcohol	11	27

**Table 5:** Method precision data for spiked sample.

Sample number	Acetone	Isopropyl alcohol
1	5242	6391
2	5346	6538
3	5369	6542
4	5289	6469
5	5240	6315
6	5281	6413
*Mean*	*5294*	*6445*
*% RSD*	*1.0*	*1.4*

**Table 6:** Accuracy data of acetone and isopropyl alcohol.

Spike level		% recovery and relative standard deviation of acetone and isopropyl alcohol	
		Acetone	Isopropyl alcohol
Level-1 LOQ	Mean % RSD	108.2 2.6	109.9 2.0
Level-2 LOQ	Mean % RSD	94.2 0.2	106.8 1.6
Level-3 LOQ	Mean % RSD	94.7 1.5	104.9 1.7
Level-4 LOQ	Mean % RSD	101.2 0.6	110.1 0.1
Level-5 LOQ	Mean % RSD	101.3 0.5	108.1 1.0

**Table 7:** Linearity data of acetone and isopropyl alcohol.

Description	Dipyridamole and known impurities	
	Acetone	Isopropyl alcohol
Square of correlation coefficient (*R*^2^)	0.998	0.997
Slope	1385.9	357.7
*Y*-intercept	100,186.4	39,555.8
% *Y*-intercept	1.4	2.1

**Table 8:** Ruggedness data.

Impurity	% RSD for six individual preparation	The overall % RSD (*n*=12)
Acetone	0.6	1.4
Isopropyl alcohol	0.7	3.1

**Table 9:** Robustness data.

Parameter variation	The present relative standard deviation of peak area for six replicate injections should not more than 10.0		The present relative standard deviation of retention time for six replicate injections should not more than 10.0	
	Acetone	Isopropyl alcohol	Acetone	Isopropyl alcohol
Flow 0.8 mL/min	0.4	0.6	0.0	0.0
Flow 1.2 mL/min	0.5	0.7	0.1	0.0
Column oven temperature 35°C	0.3	1.0	0.0	0.0
Column oven temperature 45°C	0.4	0.6	0.0	0.0
Headspace vial equilibration time 15 min	1.2	3.1	0.0	0.0
Headspace vial equilibration time 25 min	0.2	0.7	0.0	0.1
Headspace vial oven temperature 75°C	0.5	0.8	0.0	0.1
Headspace vial oven temperature 85°C	0.2	0.4	0.0	0.0
